# Graded activity with and without daily-monitored-walking in patients with type 2 diabetes with low back pain: secondary analysis of a randomized-clinical trial

**DOI:** 10.1186/s40945-021-00104-3

**Published:** 2021-04-15

**Authors:** Opeyemi Ayodiipo Idowu, Ade Fatai Adeniyi, Andrew Edo, Adesoji Fasanmade

**Affiliations:** 1 Department of Physiotherapy, College of Medical Sciences, School of Basic Medical Sciences, University of Benin, Benin City, Nigeria; 2 Department of Physiotherapy, College of Medicine, University of Ibadan, Ibadan, Nigeria; 3 Deparment of Medicine, University of Benin, Benin City, Nigeria; 4 Department of Physiology, College of Medicine, University of Ibadan, Ibadan, Nigeria

**Keywords:** Low back pain, Psychosocial factors, Type-2 diabetes mellitus, Graded activity

## Abstract

**Background:**

Graded activity is gradually emerging as a preferred choice in improving psychosocial outcomes including pain self-efficacy, fear-avoidance beliefs, and back-pain beliefs in the general population with low back pain (LBP). Such evidence is, however, lacking among patients with concomitant LBP and type-2 diabetes mellitus (T2DM). This secondary analysis of a randomized control trial aimed to compare the efficacy between graded activity augmented with additional daily-monitored-walking and graded activity alone on disability, pain self-efficacy (PSE), fear-avoidance beliefs (FAB), back-pain beliefs (BPB) and glycaemic control (HbA1c) in patients with concomitant LBP and T2DM.

**Methods:**

Fifty-eight patients with concomitant LBP and T2DM were randomised into two groups, graded activity with daily-monitored-walking group (GAMWG = 29) or (graded activity group (GAG = 29) in this 12-week single-blind trial. Both groups received graded activity (home/work-place visits, back school and sub-maximal exercises) while the GAMWG received additional daily-monitored-walking. Disability and selected psychosocial outcomes were assessed at weeks 0, 4, 8 and 12 using Roland-Morris disability, fear-avoidance behaviour, pain self-efficacy and back belief questionnaires. Glycaemic control was assessed at weeks 0 and 12 using a point-of-care system (In2it, Biorad Latvia). Data were analysed using mean, median, Friedman’s ANOVA, Mann-Whitney test and t-tests.

**Results:**

Participants’ mean age was 48.3 ± 9.4 years (95%CI: 45.6, 50.9) while 35.3% were males. The GAMWG participants (*n* = 25) had better outcomes (*P* < 0.05) than GAG participants (*n* = 26) on PSE (1.0, 3.0; r = − 0.1) and FAB (0.01, − 2.0; r = − 0.1) at week 4, LBP-related disability (0.01, − 2.0; r = − 0.2) at week 8 and glycaemic control at week 12 (− 0.59 ± 0.51%,-0.46 ± 0.22%). No other between-group comparisons were statistically significant.

**Conclusion:**

Graded activity with daily-monitored-walking provided earlier improvements on disability, pain self-efficacy, fear-avoidance beliefs, and glycaemic control, but not back pain beliefs, in patients with concomitant LBP and T2DM.

**Trial registration:**

PACTR201702001728564; 26 July, 2016 (retrospectively registered).

## Background

Low back pain (LBP) is pain between the rib cage and the inferior gluteal folds with or without radiculopathy [1]. It is a prevalent and expensive health problem and remains a significant reason why people seek health care [1]. LBP results in significant loss of function and disability. It is categorised as being specific or non-specific. Non-specific LBP, defined as LBP of unrecognised or unidentified pathology, accounts for 90% of all LBP cases [1]. According to Hoy et al. [2], the prevalence of LBP ranges between 8 and 82.5%, and most people will experience LBP at some time during their lives. Although spontaneous recovery from non-specific LBP occurs within 3 months of onset in about 33.3% of individuals, more than 71% will have LBP 1 year after [3]. The aetiology of LBP is complex and multifaceted in that several co-occurring risk factors are linked to its onset, course and persistence. These factors include individual, occupational, and psychosocial factors [4, 5]. The psychosocial framework which postulates that patho-anatomy, psychological, socioeconomic, ecological and cultural elements all impact on the onset and persistence of LBP provides a general explanation for the influence of psychosocial constructs in the management of persistent LBP [6]. Specifically, factors including fear avoidance behaviour, pain self-efficacy, belief of consequence of back pain are known to perpetuate LBP [7, 8]. Therefore, it is essential to pay attention to these factors during rehabilitation.

Exercise interventions that encourage continued activity using a graded approach (i.e. engaging a patient in a pre-determined activity quota, in a controlled and time-bound fashion), despite attendant LBP, is gaining acceptance as an efficacious remedy for persistent LBP [9]. One such intervention is the graded activity [9]. In the graded activity, exercise activities are not determined based on pain alone, but by pre-determined activity quotas [10]. Exercise programmes that have used graded activity principles for the management of patients with LBP have reported improvement in patients’ health status, specifically reduced disability and reduced work-absenteeism [11–13]. Van Der Giessen and colleagues [12] in a systematic review recommended for further studies to substantiate the current evidence for the efficacy of graded activity. Whilst studies suggest the efficacy of graded activity in the management of LBP for the general population, there appears to be a paucity of studies conducted in well-defined LBP populations having other comorbid health problems. Such comorbidities include type 2 diabetes mellitus (T2DM) and obesity.

Musculoskeletal pain, including LBP is a known problem among patients with T2DM [14]. However, a causal relationship between the two remains unclear. One animal study suggests that T2DM in rats is associated with early intervertebral disc degeneration resulting from accumulation of advanced glycation end-products [15]. In humans, T2DM has been linked to lumbar spinal stenosis [16], a condition associated with persistent LBP and lumbar disc disease secondary to diabetes-related microangiopathy of the lumbar disks [17]. Further, persons with T2DM and metabolic derangements, such as peripheral neuropathy and obesity, have excess adipose tissues stored in their skeletal muscles [18]; leading to increased susceptibility to disc prolapse, loss of spinal flexibility and consequent LBP. As a result of these aforemention factors that could perpetuate LBP in the T2DM population, favourable results of graded activity on LBP may not be extrapolatable for those having concomitant LBP and T2DM. Lifestyle management including physical activity and medical diet therapy, are the corner stones of diabetes management [19]. Although, the effectiveness of PA intervention in the management of musculoskeletal pain of patients with T2DM is not well documented, reports favouring PA are emerging [20]. Further, PA activity counters such as pedometers has been proven to be a novel and highly useful motivator, a direct source of feedback and memory prompt and reminder to be physically active in the T2DM population [21]. Our previous study examined the efficacy of a 12-week graded activity with and without daily-monitored walking on pain intensity, static abdominal muscle endurance and static back extensors muscle endurance among patients with concomitant LBP and T2DM [22]. Participants who received graded activity with daily-monitored walking had better outcomes for static back extensors endurance at week 8, than those who received graded activity intervention alone. No other between-group comparisons (pain and static abdominal muscular endurance) were statistically significant [22]. It is however unknown whether graded activity alone would be adequate in improving LBP-related disability and psychosocial outcomes (such as pain-self efficacy, fear-avoidance beliefs and back pain beliefs) of patients with concomitant LBP and T2DM or the addition of a physical activity intervention (pedometer assessed daily-monitored-walking) usually targeted at associated derangements (such hyperglycaemia, loss of flexibility, and reduced muscle strength) in T2DM would be necessary. Therefore, this secondary analysis of our previously published randomized-clinical trial (RCT) aimed to compare the efficacy of graded activity with additional daily-monitored-walking and graded activity alone on disability and psychosocial outcomes of patients with concomitant LBP and T2DM.

## Methods

We conducted a secondary analysis of an RCT which compared two groups of patients with concomitant LBP and T2DM; one receiving graded activity augmented with additional daily-monitored-walking and the other graded activity alone. Outcome measures included disability, fear avoidance beliefs, pain-sef efficacy, back pain beliefs and glycaemic control.

### Trial design and randomization

The study design adopted for this study was a 12-week single-blind randomised clinical trial involving 58 patients with concomitant LBP and T2DM. The first participant was recruited November 5, 2014 to while the last day of follow-up was July 26, 2016. Participants were randomly assigned to graded activity group (GAG) and graded activity with daily monitored walking groups (GAMWG) with both receiving hospital-based treatments, two sessions per week for the duration of the study. Patients were randomized into treatment groups by the researcher using a computer-generated random permutation blocks. Each restricted computer-generated block permutation was printed on a small coloured card and placed in a sealed opaque numbered envelope. Participants were assigned the groups represented by the block size until the block sequence was exhausted. To enter the next set of patients into the study, the researcher opened the next consecutively numbered envelope.

### Participants

The study participants were 58 male and female T2DM patients (48.3 ± 9.4 years) diagnosed with persistent non-specific LBP of not less than 3 months. They were referred for physiotherapy by an endocrinologist or an orthopaedist at Federal Medical Centre (Now Federal Teaching Hospital), Ido-Ekiti, Ekiti-State, Nigeria and University of Benin Teaching Hospital, Benin-City, Edo State, Nigeria. These two hospitals have the highest referrals for orthopaedic, and diabetes cases in their various states and their selection were based on convenience and availability of patients. Individuals who could comprehend either of English or Yoruba language, without any apparent deformities affecting the trunk or upper and lower extremities were eligible to participate in the study. Exclusion criteria for the study include having morbidities beside T2DM (like uncontrolled hypertension, stroke and asthma), additional disabling conditions such as severe neuropathy and amputations and red flags suggestive of spinal pathology. The University of Ibadan/ University College Hospital Health Research and Ethics Committee (UI/EC/13/0093) and University of Benin Teaching Hospital Health Research and Ethical Committee (ADM/E22/VOL.VII/1187) gave approvals for the study. Participants gave their written informed consent after the research aims and protocols were explained to them.

### Interventions

Participants in the GAG received graded activity only while those in the GAMWG received graded activity with daily-monitored-walking. The graded activity protocol comprised functional assessments; work-place and a home visit, and interventions including back school and an individual, sub-maximal, gradually increased exercise programme. In addition, cognitive behavioural principles including positive reinforcement, education on pain mechanisms and addressing mal-adaptive pain behaviours and pain-related anxiety was incorporated into the graded activity by the researcher to overcome participants’ natural anxiety associated with pain and activity [23]. The functional assessments, including the static abdominal muscular endurance, static back extensors endurance, and the six minutes-walk test have been fully described elsewhere [22]. Subsequently, each participant’s physical work demands at work and home (as applicable) was assessed in order to develop an individualised graded activity. The assessments were used to reinforce the need for adjustments during the initial and subsequent back schools. The work-place and home visits were undertaken thrice before the end of the study, namely: at the commencement of the individually graded activity programme, at week four as well as week eight of intervention. The work-place and home visit was conducted by the principal researcher (OI), an orthopaedic physiotherapist.

The principal researcher also taught the patients individually the main content of the Nigerian Back School [24]. The Back School recommended by Lindstrom et al. [25] had to be replaced with the Nigerian Back School because the majority of items included were not culturally adaptable to the research context. Participants were first taught the back school contents during the first home visit. Subsequently, other sessions were during the treatment sessions. The duration of the back school was 10 min per session. During the back school, participants were educated on pain mechanisms, and how to address mal-adaptive pain behaviours and pain-related anxiety. The exercise component of the graded activity was then administered to participants following the protocol described by Lindstrom et al. [25]. This exercise component was delivered in the exercise gymnasia of the physiotherapy departments of Federal Medical Centre, Ido-Ekiti, Ekiti-State, Nigeria and University of Benin Teaching Hospital, Benin-City, Edo State, Nigeria. The intervention consisted of a 1-h exercise session (warm up-5 min, bicycle ergometry- 20 min, cool down-5 min, other exercises 20 min) which participants attended twice per week for 12 weeks. According to the graded activity principle, the exercise goals were pre-set. New exercise targets for each participant were reviewed by the researcher at the end of weeks 4 and 8. The exercise progression was according to individual participant’s ability to meet up with the pre-set exercise goal. Positive reinforcement in terms of verbal encouragements was provided each time the patient achieves the pre-determined quota. Following the graded activity principle, the pre-set exercise intensities were the minimum desirable exercise quota, and the participants had to perform the exercises each session according to this quota. Each participant was encouraged to achieve the minimum desirable quota. However, those who can go beyond this were allowed to go at their coping intensity. The graded activity intervention was individualized and delivered to participants face-to-face. Details of the home and work-place visits, back school and the exercise components of the GA are contained in Table 1.

**Table 1:** Details of the Graded Activity Protocol

Pre Exercise Component	Description	Dosage/ Progression/Frequency
1. 1. Home and work place visit	Researcher’s assessment of each patient’s physical work demands in terms of requirements for standing, standing and twisting, walking, sitting, sitting and twisting, lying, lying and twisting, kneeling, squatting, forward bending, backward bending, working with the arms above the shoulders, working with the hands above the shoulders, and working with the hands and arms without support.	Carried out at weeks 0, 4 and 8 of the study.
2. 2. Back School	Patient taught the main content of the Nigerian Back School [23]. Contents included details of basic anatomy, functions of the muscles, functions of the back, and LBP disability treatments. Emphasis was placed on the body’s natural capacity for healing. Observed individual working postures and working techniques, both in the work place and at home were discussed in terms of biomechanical load. The advantages of PA and the damaging effects of poor posture and immobilization on muscles, tendons, joints, and discs were emphasized.	Carried out for 10 min during each treatment session at weeks 1 through to week 12 of the study.
**Exercise Component**		
1. Warm up	Comprised stretches and strolling at self-determined pace around the research venue.	5 min
2. Aerobic training	Participants pedalled a bicycle ergometer (American fitness, Model YK-B28N) at an intensity of 50–80% of Heart Rate Reserve (HRR)	Pre-set baseline, week 5–8, and week 9–12 exercise goals set at: 50, 70 and 80% of HRR, respectively.
3. Abdominal sit up exercises	This was performed with the patient in supine lying; knees flexed, feet unsupported, hands stretched toward the knees. The trunk was then curled until the back has no support.	Pre-set baseline, week 5–8, and week 9–12 exercise goals set at: 1 set of 7–10 repetitions, 2 sets of 7–10 reps, and 3 sets of 7–10 reps, respectively.
4. Dynamic back extension exercise	With the patient lying prone, arms along the trunk, the trunk was raised until there was no contact between the chest and the support surface.	Pre-set baseline, week 5–8, and week 9–12 exercise goals set at: 1 set of 7–10 repetitions, 2 sets of 7–10 reps, and 3 sets of 7–10 reps, respectively.
5. Bent over row-dumb bells exercises	With two dumb bells held one in each hand, the patient bending forward through the hips, trunk upright knees slightly flexed and the dumb bells held hanging down by the side, patient was asked to flex the elbows while forearms were still held firmly to the trunk and thereafter extended the elbows.	One Repetition Maximum (1-RM) was determined by the Bryzcki’s formula (Bryzcki, 1993). Pre-set baseline, week 5–8, and week 9–12 exercise goals set at: 1 set of 7–10 repetitions, 2 sets of 7–10 reps, and 3 sets of 7–10 reps, respectively.
6. The squatting exercise	With the normal lordotic posture and an erect spine still maintained, patient was asked to flex the knees to a point where the tops of the thighs were parallel to the floor.	Pre-set baseline, week 5–8, and week 9–12 exercise goals set at: 1 set of 7–10 repetitions, 2 sets of 7–10 reps, and 3 sets of 7–10 reps, respectively.
7. Cool-down phase	Low intensity exercise and stretches	Five minutes.

Participants in the graded activity with daily monitored walking in addition to graded activity had an objectively daily monitored walking programme using a (Omron Walking style III HJ-203-EG) pedometer. Participants were instructed to achieve the daily recommended level that is beneficial for health and wellbeing based on the “5,500 daily steps or 4,600 steps per day if averaged over a week of free-living behaviour” recommendation for patients with chronic illness [26]. Participants were instructed to take at least 5500 steps per day. Participants who may have been achieving 5500 steps per day prior to the research were encouraged to achieve more number of steps as their coping intensity would allow. They were taught on possible ways of achieving this number of steps. These include the use of public transport, and not personal vehicles, from home to their places of work and worship, and stopping the use of transport for walkable distances. To ensure participants’ compliance with the pedometer-based walking activity, four text messages per day were sent every four hours (between 7.00 am to 7.00 pm) to remind them to engage in sufficient walking activity. Pedometer step counts were collected and used as an index to monitor adherence to the walking programme. This multi-location study was carried out at the exercise gymnasia of the Department of Physiotherapy, Federal Medical Centre, Ido-Ekiti, Ekiti State, and the Department of Physiotherapy, University of Benin Teaching Hospital, Benin-City, Edo-State, Nigeria. Further, home-based daily-monitored-walking was completed by participants in their homes.

### Outcomes

Two (blinded) research assistants (physiotherapists) coordinated recruitment, eligibility screening, assignment of the patients into the treatment groups and measurement of outcomes. A biodata form was used to document socio-demographic (age; gender-male, female; marital status-single, married, divorced, widowed; educational level; primary school, secondary school, polytechnic and university occupational status; unemployed, employed, retired) of each participant. Also, the height and weight of participants were measured using a height-weight meter (ZT-160, China). Body mass index of participants was calculated using standard procedures. In addition baseline physical activity of participants was profiled using the International Physical Activity Questionnaire (IPAQ) [27]. The IPAQ was administered and scored following standard procedures.

The 24-item Roland Morris disability questionnaire (RMDQ) was used to assess LBP-related disability of participants [28]. The RMDQ was used to assess how LBP affected participants’ activities of daily living including housework, moving around, self-care, walking, sleeping, sitting, irritability and appetite. Each participant was asked to select the item that applies to the activity affected by LBP. One point was awarded for each selected item. The selected items were summed up to determine the disability score. A total maximum score of 24 signifies the highest possible disability level and 0 means that there is no disability. The Fear Avoidance Beliefs Questionnaire (FABQ) was used to assess the avoidance behaviour of participants [29]. The FABQ was developed by Waddell et al. to investigate the fear-avoidance behaviour among LBP patients in the clinical setting [29]. The questionnaire consists of 2 subscales; the Physical Activity subscale (FABQPA) with items 1–5 and the Work subscale (FABQW) with items 6–16. Each subscale is graded separately by summing the responses of the scale items (0–6 for each item). The total FABQPA subscale score (Minimum = 0, Maximum = 24) was obtained by the summation of items 2, 3, 4 and 5 scores while the total FABQW subscale score (Minimum = 0, Maximum = 42) was obtained by the sum of scores of items 6, 7, 9, 10, 11, 12, and 15. Higher scores on the FABQ are indicative of greater fear and avoidance beliefs.

The 10-item pain self-efficacy questionnaire (PSEQ) was used to assess the pain self-efficacy belief of participants. It covers a range of functions including household chores, socialising, work as well as coping with pain without medication. Responses for each item were on a 7 point Likert scale, where 0 = not confident at all and 6 = completely confident. A total score ranging from 0 to 60 was calculated by adding the scores for each item with higher scores reflecting stronger self-efficacy beliefs [30]. The back beliefs questionnaire (BBQ) was used to assess the participants’ beliefs about the consequences of back pain. The questionnaire consisted of 14 statements to which the respondents indicated their level of agreement on a 5-point Likert scale. A score of 1 indicates complete disagreement and a score of 5 complete agreement. As 5 of the 14 statements are distractors, the scores of the nine remaining statements were reversed and then summed to provide a total score ranging from 9 to 45. A lower score indicated that the respondent had more negative beliefs about back pain [31].

The Visual analogue scale (VAS) was used to assess the worst pain intensity of participants [32]. The VAS represents the intensity dimension of pain by a 100 mm line with two anchors of “no pain” and “worst pain I ever felt”. Each patient was asked to mark the point on the 10 cm line, which best described his or her worst pain level. The distance in centimetres between the first anchor “no pain” and the point marked by the patient was documented as the intensity of back pain felt. Results and discussion on the comparative efficacy of GAG and GAMWG on pain intensity has been reported elsewhere [22]. Further, glycated haemoglobin (a measure of glycaemic control in the past 3 months) [33] was assessed using a point-of-care system (In2it, Biorad Latvia). The assessment of glycated haemoglobin (HbA1c) was necessary as previous studies have suggested a positive association between HbA1c and musculoskeletal disorders [34, 35]. The RMDQ, FABQ, PSEQ and BBQ were translated and validated in the Yoruba language. The RMDQ (α = 0.93, Intra Class Correlation (ICC): 0.99), FABQ (α =0.9; ICC = 0.97; 0.94–0.97), PSEQ (α = 0.79, ICC = 0.86) and BBQ (α = 0.71, ICC = 0.79) yielded excellent construct validities and test-retest reliabilities. Outcomes including LBP-related disability, pain self-efficacy, fear-avoidance behaviour and back pain beliefs were obtained and recorded at baseline at participant’s first appearance and also at the end of weeks 4, 8 and 12 of the study. The language choice in the administration of the tools (Yoruba or English) was based on participants’ preference and language comprehensibility. Investigations of glycaemic control (HbA1c) of participants were done only at baseline and at week 12 of the study. The researcher kept a record of the number of treatment visits made by each researcher in a diary to assess the adherence to the graded activity programme.

### Sample size

Using the Cohen’s table [36] with the effect size of 1.0, 80% power, degree of freedom of 1, α level of 0.05, seventeen participants per group (based on within-group effects) was found adequate for the study. According to Cohen [36], 4 patients (10%) were added to the study population to make room for attrition (i.e. 34 + 4). However, a total number of 58 participants were recruited into the study.

### Statistical methods

Mean and standard deviation was used to summarise continuous variables. Median and interquartile range was used to summarise data that did not follow a normal distribution. Frequency and percentage were used to summarise categorical variables. Friedman’s Analysis of Variance (ANOVA) was used to compare within-group differences in LBP-related disability, pain self-efficacy, fear-avoidance behaviour and back pain beliefs of patients for GAG and GAMWG across weeks 0, 4, 8 and 12 of the study. Post hoc analysis of Wilcoxon signed ranked test was used for the within-group analysis. Mann-Whitney U test was used to compare between-group differences in the mean changes of outcomes of patients. Effect size (r) was calculated for between group analysis using the formula *r* = *Z*/ √ 2 where Z = the standardized value for the U-value and n = total number of observations on which Z is based [37]. Further, for within group analysis, effect size (W) was calculated using *W* = χ2 /N (k-1) where χ2 = Friedman test statistic value, N = sample size and k = number of measurements per test [37]. An effect size of 0.2, 0.5 and 0.8 was adjudged to be small, medium and large, respectively [37]. Unpaired t-test (including effect size d = mean difference between the two groups/pooled standard deviation [38]) was used to investigate differences in the mean change of glycaemic control of participants between the two groups with the level of significance set at α = 0.05.

## Results

**Fig. 1:**
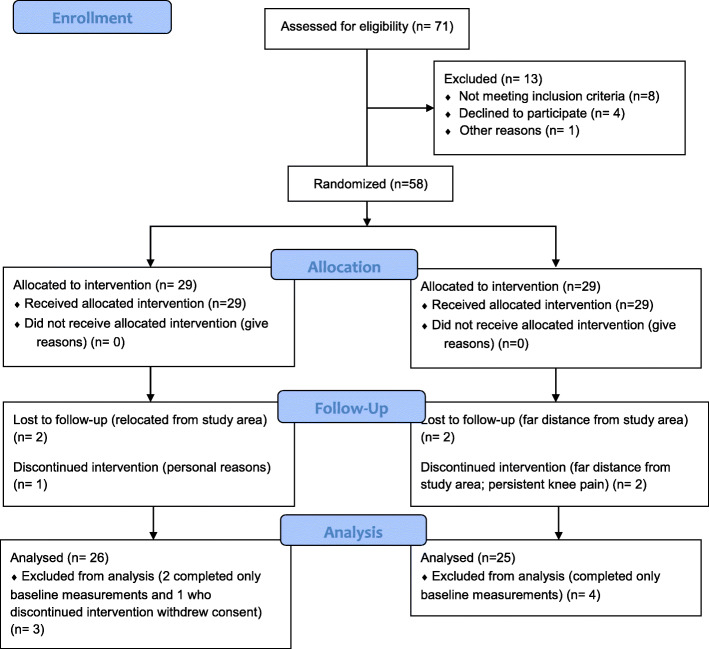
Consolidated Standards of Reporting Trials (Consort) flow diagram for the recruitment of participants

Fifty-eight patients with concomitant LBP and T2DM who met the inclusion criteria for the study were assessed and randomly allocated into one of two groups (graded activity group (GAG), *n* = 29, or graded activity with daily-monitored-walking group (GAMWG), n = 29). The Consolidated Standards of Reporting Trials (CONSORT) flow diagram for the recruitment of participants is shown in Fig. 1.

The mean number of treatment visits between the GAMWG (23.44 ± 1.58) and GAG (23.88 ± 0.59) was not significantly different (*P* = 0.49). The mean age of GAMWG participants was 48.28 ± 9.41 years, 32% were men. Also, the mean age of GAG participants was 48.27 ± 9.56 years, 39% were men. Socio-demographic characteristics and baseline general characteristics of participants are presented in Table 2. In addition, The HbA1c of participants in GAMWG and GAG at baseline was 6.33 ± 0.90% and 6.31 ± 0.87%, respectively.

**Table 2:** Socio-demographic and baseline general characteristics of all participants by treatment group

**Variable**	**GAMWG**^**a**^ **n (%)**	**GAG**^**b**^ **n (%)**	**Total (*n*** **= 51)** **n (%)**	
**Gender**				
Male	8. (32%)	10 (38.5%)	18 (35.3%)	
Female	17. (68. %)	16 (61.5%)	33. (64.7%)	
**Marital Status**				
Married	24. (96%)	25. (96.2%)	49 (96.1%)	
Widowed	1. (4.%)	1. (3.8%)	2 (3.9%)	
**Education**				
Primary School	6 (24%)	0 (0%)	6 (11.8%)	
Secondary School	6 (24%)	6 (23.1%)	12 (23.5%)	
Polytechnic	2 (8%)	2 (7.7%)	4 (7.8%)	
University	11 (44%)	18 (69.2%)	29 (56.9%)	
**Occup. Status**				
Unemployed	3 (12%)	4 (15.4%)	7 (13.7%)	
Employed	17 (68%)	18 (69.2%)	35 (68.6%)	
Retiree	5 (20%)	4 (15.4%)	9 (17.7%)	
**Variable**	**GAMWG**^**a**^ **Mean ± SD**	**GAG**^**b**^ **Mean ± SD**	***t-value***	***P*-value**
Age (years)	48.28 ± 9.41	48.27 ± 9.56	−0.00	0.99
BMI (Kg/m^2^)	26.48 ± 3.62	27.32 ± 2.22	1.00	0.31
HBA1c	6.33 ± 0.90	6.31 ± 0.87	0.10	0.92
Physical Activity (MetMin/day)^c^	1359.49 ± 635.0	1434.87 ± 1028.47	0.32	0.75
	**Median (IQR)**	**Median (IQR)**	**U-value**	***P*-value**
VAS scores (cm)	6.94 (0.15)	7.01 (0.20)	299.50	0.62
LBP disability Scores	9.0 (3.50)	9.50 (6.50)	308.00	0.75
Pain Self-Efficacy	38.0 (9.50)	37.0 (6.50)	315.00	0.85
Fear avoidance beliefs	38.0 (9.0)	37.0 (11.50)	311.50	0.80

*GAG*-Graded activity group; *GAMWG* - Graded activity with daily –monitored-walking group; *Occup.* – Occupational; ^a^ - n = 25, ^b^ - n = 26; *SD*- Standard deviation; *BMI*-Body mass index; *HBA1c*- Glycated haemoglobin; *VAS*-Visual analogue scale; *IQR*-Inter-quartile range. ^c^ – Assessed with the International Physical Activity Questionnaire

Participants in the GAMWG had significantly lower LBP-related disability scores at week 8 of the study; however, there was no significant difference in the LBP-disability scores of participants in GAMWG and GAG at weeks 4 and 12. Further, participants in the GAMWG had significantly higher pain-self efficacy scores, and lower fear-avoidance belief scores than participants in the GAG at week 4; but no such significant differences were found at weeks 8 and 12 of the study. There was no significant difference in the back belief scores between GAMWG and GAG participants at weeks 4, 8, and 12 of the study. Also, the results showed a statistically significant difference (d = − 1.1, 95%CI = -1.7 to − 0.5, *P* = 0.001) in the change scores of HbA1c between GAMWG (− 0.59 ± 0.51%) and GAG (− 0.46 ± 0.22%). Table 3 shows the results of within-group analysis for LBP-related disability, pain-self efficacy, fear-avoidance beliefs and back-pain beliefs in the GAMWG and GAG, respectively while comparison of LBP- disability scores and the selected psychosocial outcome variables between participants in GAMWG and GAG at weeks 4, 8 and 12 of the study are presented in Table 4. Patients did not report any serious adverse event throughout the duration of the intervention. There were ocassional reports of muscle soreness which resolved within 24 h.

**Table 3:** Within-group comparisons of participants’ disability scores, psychosocial outcomes across the 4-time points of the study

Variable	Groups	Time frame				***W***	χ2	***P***^**ѱ**^
		Week 0	Week 4	Week 8	Week 12			
		Median (IQR)	Median (IQR)	Median (IQR)	Median (IQR)			
LBP disability scores	GAMWG	9.0 (3.5)^a^	7.0 (3.0)^b^	5.0 (3.0)^c^	4.0 (1.5)^d^	0.93	69.8	< 0.001*
	GAG	9.5 (6.5)^a^	8.0 (5.3)^b^	7.0 (4.0)^c^	5.0(3.0)^d^	0.74	58.0	< 0.001*
Pain-self-efficacy	GAMWG	38 (9.5)^a^	42 (6.0)^b^	44.0 (6.0)^c^	50 (8.0)^d^	0.90	67.6	< 0.001*
	GAG	37.0 (6.5)^a^	38.0 (5.0)^b^	40.0 (8.5)^c^	45.0 (6.5)^d^	0.87	68.2	< 0.001*
Fear-avoidance beliefs	GAMWG	38.0 (9.0)^a^	37.0 (9.5)^b^	27.0 (6.0)^c^	22.0 (6.0)^d^	0.96	72.0	< 0.001*
	GAG	37.0 (11.5)^a^	37.0 (9.0)^b^	30.50 (4.8)^c^	24.0 (7.0)^d^	0.98	76.0	< 0.001*
Back-pain beliefs	GAMWG	28.0 (7.5)^a^	30.0 (7.5)^b^	32.0 (2.5)^c^	35.0 (3.5)^d^	0.96	72.0	< 0.001*
	GAG	28.5 (6.5)^a^	30.0 (6.3)^b^	30.5 (4.0)^c^	33.0 (4.0)^d^	0.93	72.3	< 0.001*

IQR-Inter-quartile range; *W*-Effect size (Kendall’s *W*); *- Significance at α = 0.05; ^a b c d^ - post-hoc indicates that values with different superscript are significantly (p < 0.05) different; values with the same superscripts are not significantly (*p* > 0.05) different. Ѱ = Friedman’s ANOVA and Wilcoxon signed rank test post-hoc test was used for within-group comparison

**Table 4:** Comparison of changes in low back pain disability scores and selected psychosocial outcome variables between participants in GAG and GAMWG at weeks 4, 8 and 12 of the study

Variable	Time Frame	GAMWG (n = 25) Median (IQR)	GAG (n = 26) Median (IQR)	***U-value***	r	***P***-value
LBP disability	Weeks 0–4	−2.0 (1.5)	−2.0 (1.0)	284.0	0.01	0.42
	Weeks 4–8	−2.0 (2.5)	0.01 (2.0)	157.5	0.20	0.00*
	Weeks 8–12	− 2.0 (1.5)	−1.50 (1.0)	315.0	0.001	0.85
Pain self-efficacy	Weeks 0–4	3.0 (5.0)	1.0 (2.0)	203.0	0.1	0.02*
	Weeks 4–8	2.0 (2.0)	1.50 (1.5)	252.5	0.04	0.16
	Weeks 8–12	6.0 (5.0)	4.0 (2.3)	237.0	0.05	0.10
Fear avoidance beliefs	Weeks 0–4	−2.0 (5.0)	0.01 (2.0)	210.0	0.10	0.02*
	Weeks 4–8	−9.0 (4.0)	−7.0 (5.0)	251.0	0.04	0.16
	Weeks 8–12	−5.0 (4.0)	−5.50 (5.3)	310.0	0.02	0.78
Back pain beliefs	Weeks 0–4	2.0 (1.0)	1.0 (3.0)	283.0	0.01	0.42
	Weeks 4–8	2.0 (3.0)	1.0 (3.3)	246.5	0.04	0.13
	Weeks 8–12	3.0 (2.0)	3.0 (2.0)	299.0	0.005	0.62

*GAG*-Graded activity group; *GAMWG*-Graded activity with daily monitored walking group; r-Effect size; IQR-Inter-quartile range; *- Indicates significance at α = 0.05

## Discussion

This current study is a secondary analysis of a previously published RCT. We compared the efficacy of combined graded activity and daily-monitored-walking with graded activity alone on disability, pain-self efficacy, fear-avoidance beliefs and back pain beliefs in patients with concomitant LBP and T2DM. We found that graded activity with daily-monitored-walking led to better outcomes than those in the graded activity group in terms of earlier improvements on pain-self efficacy and fear avoidance beliefs at week 4, and LBP-related disability scores at week 8. The interventions were safe to administer as participants were not inflicted with any serious injuries other than ocassional muscle soreness which did not last for 24 h. The earlier improvements on LBP-related disability, pain-self efficacy and fear-avoidance beliefs found among the patients in the graded activity with GAMWG may have been as a result of the additional aerobic exercise provided by the daily-monitored walking intervention.

Low back pain is a multifaceted and complex health problem which leads to disability, physical deconditioning, reduced muscular endurance and weakness of the trunk flexors and extensors [39]. These deconditioning and associated problems are reversible through general and specific exercise regimens. However, changes in impairment status or physical function are not exhaustive in explaining changes in the clinical condition of patients with persistent LBP [40]. Psychosocial factors of LBP including the fear of pain, fear of work-related activities, fear of movement assumed to cause (re) injury and pain beliefs contribute to the perpetuation of disability in individuals with LBP [41]. These psychosocial problems are likely to improve upon engaging in movement-related activities regardless of attendant pain. Moreso, patients with LBP whose treatment regimen (such as graded activity) do not circumvent pain and movements have reduced disability [42]. This phenomenon can be explicitly explained by the fear-avoidance belief model [43] which postulates that people who effectively confront their pain or fear of pain and increase their PA levels regardless of pain, will have reduced fear of pain, decreased disability, and better recovery. On the other hand, those who responded to their pain via avoidance responses are prone to more disabling persistent symptoms. Psychosocial factors are capable of playing prognostic, treatment effect modifier, mediator, or combined roles during LBP physiotherapy interventions [44]; thus, it is not surprising that the bio-psychosocial approach to LBP management is gaining much attention [45]. However, the notion of unequivocal acceptance of the bio-psychosocial approach towards LBP management by health care professionals is still evasive as a majority of LBP caregivers favour the pathoanatomic paradigm [46]. It may, therefore, be essential to keep educating caregivers on the advantages of adopting the bio-psychosocial perspective towards the management of persistent LBP.

Whilst the exercise components of graded activity may be insufficient to produce a significant change in the HbA1c of patients with T2DM as implied by the non-significant decrease in the HbA1c among participants in the GAG, the extra aerobic exercise in form of daily monitored walking may have indirectly improved the outcomes among GAMWG participants via its influence on the HbA1c. Further, as walking exercise requires muscle power in the lower extremities and spine area and contribute to mobilisation and developed strength [47], this may have resulted in reduced disability levels and subsequently better pain beliefs. Also, walking exercise performed as aerobic exercise increases the production of endorphins which binds to the opiate receptors in the pain control system of the brain and spinal cord to decrease the perception of pain [48]. Thus, such individuals may be able to engage in more PA and be able to debunk maladaptive beliefs that more movement will cause more pain. Thus, we opine that daily monitored walking may afford the patients more opportunity to be physically active, increase the confidence in their “healing back”, and help take care of their psychosocial problems.

In our study, graded activity with daily monitored walking yielded better improvements than graded activity on pain-self efficacy and fear avoidance beliefs at week 4, and LBP-related disability scores at week 8. From a clinical point of view, early improvements portend a faster remission of persistent LBP to graded activity with daily monitored walking in these patients. This might be useful in clinical decision making in the early course of treatment in patients with persistent LBP [49]. It is however important to note that the treatment effect sizes were small. It is possible that the heterogenous phenotypes of the persistent LBP of participants in this study may have been responsible for the small effect size reported. Hancock and Hill suggested that interventions for LBP should be targeted at clinically important subgroups to have better chances of identifying more effective interventions for LBP [50]. Comparing graded activity with daily monitored walking and graded activity, findings from our study showed that disability, fear avoidance beliefs and pain-self efficacy did not differ at week 12, and back beliefs did not differ at any point of the study. It is possible that at these time points (week 12 for disability, fear avoidance beliefs and pain-self efficacy; weeks 4, 8 and 12 for back pain beliefs), graded exercise alone might have provided overload stimulus to the LBP musculature and also improved the aerobic capacity of participants such that it matches that offered by graded activity with daily monitored activity.

Our findings may have some clinical implications. For optimum LBP outcomes and improved glycaemic control, additional prescription of daily monitored walking may be necessary. Also, further studies on the effects of graded activity and other commonly used LBP interventions in this population are warranted. In sum, there is no clear pattern between the effects of graded activity with or without daily monitored walking on the disability, FAB, PSE and BPB. However, it seems that a shorter period than explored in this study may lead to significant difference in the effects of the two interventions on disability, PSE and FAB.

The study findings should be interpreted with respect to study limitations. Our study assessed the short term effects of graded activity with and without daily monitored walking; thus, results should be treated with caution. For flexibility and inclusiveness, we did not have a standardised time of day for the exercise component of the graded activity; accordingly, the extent to which participants complied with the requirement of non-involvement in other exercise programs while the study lasted could not be ascertained. Also, this study is a secondary analysis of an RCT with the main findings already published. Finally, registration for this clinical trial was not done prospectively as it was not a prerequisite in Nigeria for trials as at the time the study was conducted.

## Conclusions

The findings of this study suggest graded activity with daily monitored walking yielded better improvement in LBP-related disability at week 8, pain self-efficacy and fear-avoidance behaviour at week 4, and glycaemic control at week 12. Also, there were no difference between the effects of graded activity with daily monitored walking and graded activity only for back pain beliefs at all time points as well as for the all other outcomes at week 12 of the study.

## Data Availability

All data generated or analysed during this study are included in this published article [and its supplementary information files].

## Abbreviations

LBP: Low back pain
T2DM: Type 2 diabetes mellitus
GAG: Graded activity group
GAMWG: Graded activity with daily-monitored-walking group
RMDQ: Roland-Morris disability questionnaire
FABQ: Fear avoidance beliefs questionnaire
FABQPA: Fear avoidance beliefs questionnaire-physical activity subscale
FABQW: Fear avoidance beliefs questionnaire-work subscale (PSEQ)
BBQ: The back beliefs questionnaire
HbA1c: Glycated haemoglobin
ANOVA: Analysis of Variance
